# Editorial Chit Chat

**Published:** 1869-10

**Authors:** 


### EDITORIAL CHIT CHAT.

**Near-Sightedness Among Compositors.**

M. Kohn, of Paris, has been examining the eyes of 132 compositors in printing offices. Half of them were near-sighted. This state of affairs is caused by reading badly written manuscript, and by setting type by artificial light.

**Thanks.**

To the Corning *Journal*, Wellsboro *Agitator* and Williamsport *Bulletin*, for favors received. They are all first-class weeklies, and conducted in a manner that affords one real pleasure in reading them.

**Popular Lectures.**

Now that the season has arrived when Associations make their arrangements for the winter lectures, let us have fewer speakers upon politics, less of those finely polished, and exceedingly elegant, oratorical displays, and more lectures of instruction upon scientific subjects; so that when we attend a lecture, we can say that we have learned something—added something to our store of knowledge—eh?

**To Subscribers.**

We send out but very few free copies of the BISTOURY this quarter. Our paying subscription takes nearly all of our edition. Those who want our journal in future, must pay for it. We do not blush a particle in telling you so, for we know we give you many times more than the worth of your money. Just look through the present number and see whether you don't think so too.

**Swedish Railroads.**

In Sweden, every railroad train has a complete pharmacy and medical staff, so that in case of accident the wants of the wounded are immediately attended to. Now, we think Mr. Director Fisk would do well to dispose of that big diamond of his, and numerous other *private* luxuries, and invest the proceeds in some such arrangement for the wounded upon his road, or devise means for a double track upon the Delaware Division, where such terrible accidents as at Carr's Rock and Mast Hope are constantly occurring.

**Not Any.**

We have received about a bushel of patent medicine advertisements, with orders to publish them in the BISTOURY. Excuse us, gentlemen, we are just now engaged in exposing all sorts of humbuggery and quackery, so that your cards would not look well in our columns. Nothing will be admitted in the BISTOURY that does not possess real merit and value—therefore no patent medicine advertisements of any sort will be accepted.

**Masonic Temple.**

Several propositions are being considered by the Masonic fraternity of this city to build a handsome temple—one that will be an ornament to our city and a credit to the craft. Three sites are offered; Luther Caldwell's lot, corner of Baldwin and Market streets, Mr. Wyckoff's, corner of Water street and Railroad avenue and Mr. Beardsley's, corner of Lake and Market streets. Any one of them will make a convenient place for the meetings of the several bodies. We have two Lodges, a Chapter, Council, and Commandery, in this city—all of them large bodies—requiring more spacious quarters than are now assigned to them. Let us have the temple!

THE STATE FAIR.—Many thousand people congregated in our city during fair week, and contibuted many thousand dollars to the managers of a concern which they are pleased to call "The N. Y. State Agricultural Society." Said "Society" is a humbug—a first class swindle—a contemptible fraud, and the very worst managed one to, we were ever guilty of patronizing. The managers don't understand their business, else they would have had an exhibition worthy of visitation. We are sorry that so many strangers were enticed to our city to be bamboozled by these money-grasping managers but assure them that the citizens of Elmira are as much disgusted with the affair as they possibly can be, and are in no way responsible for the failure.

Come to our County fair next year and we will give you an exhibition worthy of patronage.

**Business Advertisements.**

The BISTOURY circulates in every State and Territory of our government, rendering it a valuable means of advertising. Our leaves are nicely trimmed and cut, so that advertisements are not thrust one side where they are never seen. Parties desirous of having their cards sent all over the United States, can be accommodated by addressing the BISTOURY.

---

**Female Doctors.**

The Swedish Faculty of Medicine is about to open its doors to female doctors, and to issue diplomas to them of the same grade as those given the men. This is as it should be. We see no reason why a regularly educated female physician should not be entitled to all the privileges of the male portion of the profession. If the ladies don't make better practitioners of medicine than nine-tenths of the men engaged in the profession, we feel sorry for them. Let us have the ladies among us by all means—they will make excellent physicians and nurses, we are sure, and will add honor and dignity to the profession.

---

**John Chinaman.**

Won't some Elmirian, with a speculative turn of mind, import a few hundred Chinamen to this city, to act as "men of all work," in our kitchens? They are said to make most excellent cooks, and excel in washing and ironing. We agree to hire, at good wages, the first John Chinaman that applies for a situation in any kitchen in this city, and can find places for at least a dozen more, without half trying. We are disgusted with the class of girls that now do our kitchen work, and will hail with pleasure a Chinaman, or any other man, that will take their places.

---

**A New Uterine Supporter.**

We have had an opportunity of applying Dr. L. A. Babcock's Silver Uterine Supporter, in a distressing case of prolapsus uteri, and are known to other cases in which it has been applied, and are so pleased with the result, that we unhesitatingly recommend it to the profession as the very best instrument for prolapsus, retroversion and anteversion of the uterus, that we have ever seen. It acts promptly and most effectually, and is the only instrument we have ever yet tried that will affect the purpose for which it is intended. Dr. Babcock's address is Freeport, Ill., and the price of his supporter—$25. We take pleasure in referring physicians and others interested, to him.

**Skating Rink.**

A proposition is on foot to build a magnificent skating rink in this city, to cost about $20,000, and of the Hervey Brothers' patent. These rinks are delightful, and afford a constant source of amusement and exercise for old and young. Our young folks are in need of just such a place in which to spend the winter evenings, where they can engage in this fascinating, exhilirating and healthful exercise, shielded from the cold blasts of wind without. The rink is so constructed that spectators can sit comfortably by a warm stove, in a well lighted gallery, and look upon the happy throng of skaters below. Fathers and mothers can accompany their little ones and watch them in their merry pranks upon the ice, while all are properly shielded from cold or exposure.

We earnestly hope our monied men will take sufficient stock in the rink to assure the building of it, for just such a place is really needed in Elmira. Let us not be behind our sister cities in affording places of amusement and healthful exercise for our young folks. Mr. R. G. Hervey, a gentleman who is thoroughly familiar with the building and management of rinks, agrees to remain with us this winter and make our rink a paying success. Let the stock be subscribed for at once.

---

**Norwegian Felted Boxes.**

Ever seen any? They're funny! We will tell you how they are made and for what purpose: Take a box about one foot square, line it with successive layers of felt, leaving a round space in the centre large enough to admit the kettle in which you cook your food; then make a thick cap with which to cover up the kettle after it is placed in the box. Now, when you desire to cook your meat or potatoes, it is only necessary to heat the kettle for a few minutes up to the requisite temperature, and then to place it in the snug hole prepared for it, cover it up, and there the cooking process will go on as long as may be desirable: You are then in no danger of burning your victuals; and when they are cooked, you can keep them warm for five or six hours.

By having a number of these boxes made so as to fit your different cooking utensils, your dinner can be prepared at no expense save the original cost of starting the fire. A splendid arrangement for hot weather—you can build a wood fire to heat your cooking apparatus with, then extinguish it and have your kitchen cool for the rest of the day.

Why don't somebody introduce the boxes in this city? We want to invest something in them.

**Newspaper Morality.**

The Chicago *Journal* thinks the *Universe*, a woman's paper published in Chicago, "might be more particular in its respect for the moral decencies."

The *Universe* has recently published some pretty plain truths with regard to the abuse of the marriage relation, and has touched upon other matters of society that it would be well for every man and woman to read. The *Journal* thinks the *Universe* is indecent in referring to such matters, yet publishes the most outrageous and disgusting cards in its own columns, that would shock a Turk's idea of morality. For instance; its columns contain cards of abortionists, of medicines to prevent conception, and for cure of private diseases, etc., etc., that had much better not be admitted to respectable newspapers.

Any journal, giving place to such vile advertisements, should be excluded from the family circle—they have as much influence in urging our youth to debauchery and lewdness as the sensational dime literature and "police gazettes" of the day.

Parents cannot be too careful of the character of reading matter they furnish their children, as their thoughts and actions are, in no small measure, controlled by what they read. And for this reason, our newspaper men, the great educators of the people, should see that no immoral or obscene articles should gain admission to their journals.

**That Abortion Case.**

Some time since, our people were shocked by the intelligence, conveyed to them through a coroner's jury, of an abortion that had been committed upon a hitherto respectable young woman of our city. Rumor fastened the guilt of the deed upon a certain doctor, who, during the investigation, fled. Many are the denunciations heaped upon the head of the supposed abortionist, while comparatively little is said of the individual that madame rumor accredits with the ruin of the fair young girl.

The investigation disclosed the fact that the victim had been absent from the city—that certain medicines, called perfumery, had been sent her by express—that she was taken suddenly ill upon her arrival at home, with flooding, pain, etc., upon which the said doctor was summoned to attend the case.

An abortion, which doubtless had commenced ere the doctor's arrival, was the result. The doctor was in ill-health—had already arranged his business matters to take up his residence in the South. Now, might not the parties really guilty of the abortion and of the ruin of the young girl, have *hired* the doctor to take his departure sooner than was his intention to do, and so become the scape goat of another party's crime?

Of course, this is a mere conjecture upon our part; but, if the testimony shows anything, it certainly exhibits the fact that the doctor was not called to the case until after her arrival at home, soon after which she was attacked with the usual symptoms of threatened abortion; it is therefore by no means certain to us that he was the author of the crime. His attributing the sickness to "polypus" might have been done with a view of screening the patient; and we really see no cause for his flight unless he was *hired* to absent himself at the inquest.

If he is guilty, he should be followed and punished to the full extent of the law; if he is not, then should the guilty ones be sought for and brought to justice, for there is no crime known upon earth so damnable as abortion.

It very rarely happens that a case of criminal aboriton comes under the eye of the law, yet hundreds of cases occur in this city yearly; when one case *is* brought to light, in Heaven's name let us make an example of the perpetrators of the crime. Will not our authorities make further investigations in this matter?

